# Factors Influencing the Clinical Presentation of Breakthrough Pain in Cancer Patients

**DOI:** 10.3390/cancers10060175

**Published:** 2018-06-01

**Authors:** Sebastiano Mercadante, Paolo Marchetti, Arturo Cuomo, Augusto Caraceni, Rocco Domenico Mediati, Renato Vellucci, Massimo Mammucari, Silvia Natoli, Marzia Lazzari, Mario Dauri, Claudio Adile, Mario Airoldi, Giuseppe Azzarello, Mauro Bandera, Livio Blasi, Giacomo Cartenì, Bruno Chiurazzi, Benedetta Veruska Pierpaola Costanzo, Daniela Degiovanni, Flavio Fusco, Vittorio Guardamagna, Vincenzo Iaffaioli, Simeone Liguori, Loredana Palermo, Sergio Mameli, Francesco Masedu, Rodolfo Mattioli, Teresita Mazzei, Rita Maria Melotti, Valentino Menardo, Danilo Miotti, Stefano Moroso, Gaetano Pascoletti, Stefano De Santis, Remo Orsetti, Alfonso Papa, Sergio Ricci, Elvira Scelzi, Michele Sofia, Giuseppe Tonini, Alessandro Valle, Federica Aielli, on behalf of the IOPS-MS Study Group

**Affiliations:** 1 Anesthesia and Intensive Care & Pain Relief and Supportive Care, La Maddalena, 90146 Palermo, Italy; claudio.adile@hotmail.it; 2 Molecular and Clinical Medicine Medical Oncology, La Sapienza University of Rome, 00155 Rome, Italy; paolo.marchetti@uniroma1.it; 3 Anesthesiology, Resuscitation, and Pain Therapy Department, National Cancer Institute, IRCCS Foundation Pascale, 80131 Naples, Italy; a.cuomo@istitutotumori.na.it; 4 Palliative Care, Pain Therapy and Rehabilitation, National Cancer Institute, IRCCS Foundation, 20133 Milan, Italy; augusto.caraceni@istitutotumori.mi.it; 5 Palliative Care and Pain Therapy Unit, Careggi Hospital, 50139 Florence, Italy; rd.mediati@virgilio.it (R.D.M.); renato.vellucci@gmail.com or renato.vellucci@virgilio.it (R.V.); 6 Primary Care Unit, ASL RM1, 00165 Rome, Italy; massimo.mammucari@libero.it; 7 Department of Clinical Science and Translational Medicine, University of Rome Tor Vergata, 00133 Rome, Italy; silvia.natoli1@gmail.com (S.N.); marzialaz@hotmail.it (M.L.); mario.dauri@uniroma2.it (M.D.); 8 Second Medical Oncology Division, Città della Salute e della Scienza Hospital of Turin, 10126 Turin, Italy; airoldim@yahoo.com; 9 Medical Specialties Department, Oncology and Oncologic Hematology, ASL 13 Mirano, 30035 Venice, Italy; azzarello.giuseppe@gmail.com; 10 Medical Oncology Unit, Ospedale di Circolo e Fondazione Macchi Hospital, 21100 Varese, Italy; maurobandera@gmail.com; 11 Medical Oncology Unit, ARNAS Ospedale Civico Di Cristina Benfratelli, 90127 Palermo, Italy; livioblasi@tiscali.it; 12 Medical Oncology, A.O.R.N. Cardarelli, 80131 Naples, Italy; cartenigiacomo@gmail.com (G.C.); brunochiurazzi@alice.it (B.C.); 13 Palliative Care Unit, SAMO ONLUS, 95131 Catania, Italy; veruska72@hotmail.com; 14 Palliative Care Unit, ASL AL, 15033 Casale Monferrato, Italy; daniela.degiovanni@libero.it; 15 Palliative Care Unit, Department of Primary and Community Care, ASL 3 Genovese, 16125 Genoa, Italy; flavio.fusco@asl3.liguria.it; 16 Palliative Care and Pain Therapy Unit, European Oncology Institute IRCCS, 20141 Milan, Italy; vittorio.guardamagna@ieo.it; 17 Abdominal Medical Oncology, National Cancer Institute, IRCCS Foundation Pascale, 80131 Naples, Italy; rv.iaffaioli@gmail.com; 18 Palliative Care and Pain Therapy Unit, Papa Giovanni XXIII Hospital, 24121 Bergamo, Italy; sliguori@hpg23.it; 19 Medical Oncology Unit, National Cancer Research Center “Giovanni Paolo II”, 70124 Bari, Italy; palermo.loredana@libero.it; 20 Pain Therapy Unit, “A. Businco” Hospital, ASL 8, 09134 Cagliari, Italy; sergiomameli@gmail.com; 21 Department of Biotechnological and Applied Clinical Sciences, Section of Clinical Epidemiology and Environmental Medicine, University of L’Aquila, 67100 L’Aquila, Italy; francesco.masedu@univaq.it; 22 Medical Oncology Unit, S. Croce Hospital, 61032 Fano-Pesaro, Italy; rodolfo.mattioli@ospedalimarchenord.it; 23 Section of Clinical Pharmacology and Oncology, Department of Health Sciences, University of Florence, 50139 Florence, Italy; teresita.mazzei@unifi.it; 24 Department of Medicine and Surgery Sciences, University of Bologna, 40126 Bologna, Italy; ritamaria.melotti@unibo.it; 25 PainTherapy, S. Croce e Carle Hospital, 12100 Cuneo, Italy, vmenardo@gmail.com; 26 Pain Therapy ICS Maugeri, IRCCS Foundation 27100 Pavia, Italy; danilo.miotti@icsmaugeri.it; 27 Medical Oncology, Azienda Sanitaria Universitaria Integrata di Trieste, 34128 Trieste, Italy; stefano.moroso@asuits.sanita.fvg.it; 28 Medical Oncology, Azienda Sanitaria Universitaria Integrata di Udine, 33100 Udine, Italy; gaetano.pascoletti@asuiud.sanita.fvg.it; 29 Palliative Care and Oncologic Pain Service, S. Camillo-Forlanini Hospital, 00152 Rome, Italy; desantis.cure@hotmail.com; 30 Pain Medicine Unit, S. Camillo-Forlanini Hospital, 00152 Rome, Italy; studio.orsetti@gmail.com; 31 Pain Relief, A.O. Dei Colli, Monaldi Hospital, 80131 Naples, Italy; alfonsopapa@libero.it; 32 Division of Medical Oncology, Department of Oncology, S. Chiara University Hospital, 56126 Pisa, Italy; sergioricci20@gmail.com; 33 Medical Oncology, Castelfranco Veneto Hospital, 31033Treviso, Italy; elvira.scelzi@libero.it; 34 Department of Palliative Care with Hospice and Pain therapy Unit, “G. Salvini” Hospital, Garbagnate Milanese, 20024 Milan, Italy; msofia@aogarbagnate.lombardia.it; 35 Department of Medical Oncology, Campus Bio-Medico University of Rome, 00128 Rome, Italy; g.tonini@unicampus.it; 36 Palliative Care, FARO Foundation, 10133 Turin, Italy; alessandro.valle@tin.it; 37 Department of Biotechnological and Applied Clinical Sciences, University of L’Aquila, 67100 L’Aquila, Italy; federica.aielli@univaq.it

**Keywords:** breakthrough pain, cancer, palliative care, supportive care

## Abstract

*Background:* The aim of this study was to identify potential variables influencing the clinical presentation of breakthrough cancer pain (BTP). *Methods:* Cancer patients with a diagnosis of BTP were enrolled. Demographic and clinical characteristics, as well as background pain and BTP characteristics were collected. Multivariate analyses were conducted to assess the correlation between BTP characteristics and the variables examined. *Results:* Data of 4016 patients were analysed. Average daily number of BTP episodes was 2.4, mean intensity was 7.5, and a mean duration was 43.3 min. A short onset BTP was observed in 68.9% of patients. In 30.5% of patients BTP was predictable. There were 86.0% of participants who reported a marked interference of BTP with their daily activities. Furthermore, 86.8% of patients were receiving opioids for the management of BTP. The average time to meaningful pain relief was 16.5 min and 70.9% of patients were satisfied with their BTP medications. Age, head and neck cancer, Karnofsky, background pain intensity, predictable and fast onset BTP were independently associated with the number of BTP episodes. BTP pain intensity was independently associated with background pain intensity, fast onset BTP, and Karnofsky. Neuropathic pain mechanism was independently associated with unpredictable BTP. Variables independently associated with a longer duration of BTP were age, place of visit, cancer diagnosis, disease-oriented therapy, background pain intensity and mechanism, and unpredictable BTP. Age, Karnofsky, background pain intensity, fast onset, and long duration of BTP were independently associated with interference with daily activity. *Conclusions:* BTP has a variable presentation depending on interdependent relationships among its different characteristics.

## 1. Introduction

Pain is a frequent symptom in cancer patients [1], mainly controlled by available analgesics [2]. However, transient flares of pain may occur. This phenomenon has been defined as breakthrough cancer pain (BTP) [3], which is associated with a negative impact on quality of life [4]. Many studies have assessed the epidemiology of BTP during the last 25 years [5], reporting variable data due to different definitions, assessment tools, and methodologies [6,7,8,9]. Although BTP has been better characterized in recent years [7,10,11,12], possible factors interfering with BTP presentation have never been explored [11,13,14].

The aim of this study was to assess the main characteristics of BTP to find potential factors influencing its presentation in a large number of patients [15].

## 2. Methods

This was a national, multicenter study that involved 32 centers. The local ethical committees approved the protocol, and written informed consent was obtained from each patient. Patients were enrolled during a period of 24 months in different settings (oncology, pain therapy, palliative care, and radiotherapy) and in different places (inpatient units, day hospitals, outpatient clinics, or palliative care).

Inclusion criteria were: age ≥18 years; diagnosis of cancer at any stage; well-controlled and stable background pain with an intensity ≤4 on a 0–10 numerical scale; and presence of BTP episodes of moderate-severe intensity, clearly distinguished from background pain. The definition of BTP was: a transitory pain exacerbation of moderate to severe intensity that occurs spontaneously or predictably [7,8,10,11], well distinguished from background pain [9,11,12,16]. Exclusion criteria were: no cancer diagnosis; unstable and/or uncontrolled background pain (>4/10); no relevant peaks in pain intensity (<5/10); and incapability to be assessed. Patients meeting the inclusion criteria were consecutively surveyed.

Age, sex, setting and place of the visit, primary tumor, extent of the disease (loco regional or metastatic), type of ongoing anticancer treatments, presence and grade of mucositis [17]; presence of oral candidiasis, presence and duration of xerostomia, and Karnofsky status were recorded. For background pain, average pain intensity (0–10) in the last week, current analgesic therapy, site and mechanism of pain; were recorded. The presence of a prevalent neuropathic pain mechanism was based on patients’ description and clinical examination. Daily opioid doses were expressed as oral morphine equivalents (OME) [2]. For BTP, mean daily number of episodes in the last week, mean intensity of pain (0–10), predictability and triggering factors, site and mechanism of pain, time to maximum pain intensity (≤10 min or >10 min), mean duration of untreated episodes, relieving factors, interference with daily activities on a 0 (none) to 3 (very much) scale, who firstly made BTP diagnosis, type and dosage of medications currently used for BTP treatment, type, intensity, duration and therapy of drug adverse reactions, and time to meaningful pain relief after taking medication, were recorded. 

## 3. Statistical Analysis

Descriptive statistics have been provided both for outcomes and explanatory variables. Frequency distributions as well as explorative univariate tests have been performed to detect feasible association and correlation pattern among variables. Accordingly, *x*^2^ tests were used for categorical variables, spearman correlations where due and point biserial correlations if dummies and continuous variables were involved. This preliminary part of the analysis used a 5% statistical significance level with no adjustment for multiple testing. Multivariate generalized linear models, i.e., Poisson, logistic and linear, have been built up to model the primary end points responses, Holmes adjusted for multiple testing. The univariate examination guided the models’ covariates choice. The count Poissonian and logistic count models underwent omnibus likelihood ratio tests. Overdispersion concern with Poisson models has dealt with carrying out *x*^2^ goodness of fit tests comparing the model performance with the corresponding negative binomial. The numerical computations have been carried out using the statistical software STATA (version 14).

## 4. Results

Data on 4016 patients were available from 4067 who were surveyed in the study period. Forty patients did not meet the inclusion criteria and were erroneously screened, or had incomplete information. Patients’ characteristics are described in Table 1. The prevalent care settings were oncology and pain therapy. Patients were mainly seen as hospital inpatients. Most patients had a metastatic disease and were receiving disease-oriented anticancer treatments. Five-hundred-seventy patients (14.2%) presented with different grades of oral mucositis: 379 patients (9.4%) grade 1; 127 patients (3.2%) grade 2; and 64 patients (1.6%) grade 3–4. Candidiasis and dry mouth were detected in 224 (5.6%) and 589 (14.7%) patients, respectively. The percentage of older patients was higher in the home care setting, with a mean age of 72.4 years (SD 12.0, *p* = 0.00). Women were more represented in hospice, whereas men were more represented in home care, day hospital, outpatient clinics, and hospital wards (*p* = 0.04). Karnofsky status was lower in the home care setting (39.7, SD 11.2), and higher in day-hospital units (73.2, SD 14.5) (*p* = 0.00).

### 4.1. Background Pain and Analgesic Regimen

The average background pain intensity in the previous week was 2.98 (SD 1.7) and pain intensity on the day of assessment was 3.0 (SD 1.83). The prevalent mechanism of background pain was mixed (*n* = 2512, 62.5%), nociceptive (*n* = 1174, 29.2%), and neuropathic (*n* = 330, 8.2%). Background pain sites were: vertebral (*n* = 1557, 38.8%), abdomen (*n* = 1239, 30.8%), extremities (*n* = 816, 20.3%); pelvis (*n* = 455, 11.3%), head and neck (*n* = 301, 7.5%). The mean dosage of opioids used for background pain was 69.4 mg/day of OME (SD 88.7).

Drugs given for background pain were: anti-inflammatory drugs (*n* = 365, 9.1%), paracetamol (*n* = 1077, 26.8%); weak opioids (*n* = 389, 9.7%: codeine *n* = 129, tramadol *n* = 260); oral morphine (*n* = 329, 8.2%), oral hydromorphone (*n* = 128, 3.2%), oxycodone (*n* = 664, 16.5%); oxycodone/naloxone (*n* = 1152, 28.7%), tapentadol (*n* = 195, 4.9%), parenteral morphine (*n* = 192, 4.8%), methadone (*n* = 43, 1.1%) transdermal fentanyl (*n* = 1102, 27.5%), transdermal buprenorphine (*n* = 121, 3.0%); other drugs (*n* = 72, 1.8%). 2749 (68.45%) patients were receiving adjuvant drugs, including benzodiazepines (*n* = 427, 15.5%), antiepileptics (*n* = 1230, 44.7%), antidepressants (*n* = 377, 13.7%), antiemetics (*n* = 362, 13.2%), laxatives (*n* = 666, 24.2%) corticosteroids (*n* = 1503, 54.7%). One-hundred-thirty-nine (3.96%) patients reported some adverse effects from the background analgesic regimen. They included constipation (*n* = 93, 66.9%), confusion (*n* = 34, 24.5%), nausea (*n* = 29, 20.9%), pruritus (*n* = 15, 10.8%), gastralgia (*n* = 12, 8.6%), vomiting (*n* = 10, 7.2%), and headache (*n* = 8, 5.8%).

### 4.2. Characteristics of BTP

**Figure 1:**
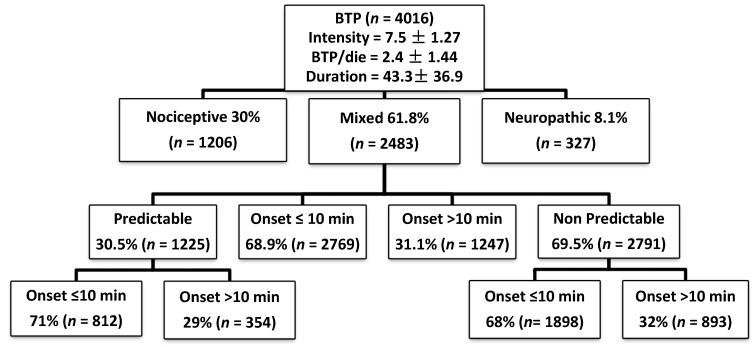
Characteristics of breakthrough cancer pain (BTP).

The characteristics of BTP summarized in Figure 1. The mean number of BTP episodes/day was 2.4 (SD 1.4, range 1–10); 64.4% of patients had 1–2 episodes/day, 29.4% had 3–4 episodes/day, and 6.2% had ≥5 episodes of BTP/day. The mean intensity of BTP was 7.5 (SD 1.3). The majority of patients (*n* = 2971, 73.9%) had an intensity of ≥7. BTP was unpredictable in 69.5% of patients. BTP sites were: vertebral (*n* = 1476, 36.7%), abdomen (*n* = 1183, 29.5%) extremities (*n* = 784, 19.5%), chest wall (*n* = 751, 18.7%), and pelvis (*n* = 401, 10%). BTP mechanism was mixed (*n* = 2483, 61.8%), nociceptive (*n* = 1206, 30%), and neuropathic (*n* = 327, 8.1%) type.

The main triggering factors for predictable BTP were activity-movement (67.4%) and swallowing (16.6%). BTP was predominantly of mixed (61.8%) or nociceptive (30%) type. Time to maximum pain intensity was ≤10 min (fast-onset BTP) in 68.9% of patients and >10 min (slow-onset BTP) in 31.1% of patients. The mean duration of untreated BTP episodes was 43.3 min (SD 36.9). 85% of patients reported that BTP limited systematically daily life. BTP interfered with daily activity: much (*n* = 2276, 56%): very much (*n* = 1127, 28.1%), a little (*n* = 542, 13.5%), nothing (*n* = 71, 1.77%), Medications for BTP were: OTCF (oral transmucosal fentanyl citrate, *n* = 130, 3.2%), FBT (fentanyl buccal tablet, *n* = 435, 10.8%), FBST (sublingual fentanyl, *n* = 570, 14.2%), FPNS (fentanyl pectin nasal spray, *n* = 807, 20.1%), INFS (intranasal fentanyl spray, *n* = 40, 1%), oral morphine (*n* = 563, 14.0%), intravenous and subcutaneous morphine (*n* = 129, 3.21%). Five-hundred-twenty-nine (13.2%) patients were not receiving BTP medications. The mean doses of FPNS, OM, FBST, FBT, OTFC, SC-M, IV-M, and INFS, were 167.7 μg (SD 125.7), 11.8 mg (SD 8.2), 231.4 μg (SD 171.1), 234.6 μg (SD 183.1), 395.4 μg (SD 280.5), 10.1 mg (SD 4.6), 8.2 mg (SD 6.1), and 100 μg (SD 50.7), respectively.

### 4.3. Adverse Effects of BTP Medications

Adverse reactions attributed to BTP medications were reported in 53 out of 2139 (2.5%) patients with available data, and were: constipation (*n* = 21), confusion (*n* = 20), nausea (*n* = 7), headache (*n* = 2), vomiting (*n* = 4) and other unspecified adverse effects (*n* = 12). The intensity was mild, moderate, and severe in 44 patients (83.0%), five patients (9.4%), and two patients (3.8%), respectively. In 33 of these patients (62.3%) no specific therapeutic changes were required, while in 11 cases (20.7%) it was deemed necessary to treat the adverse effects or discontinue the BTP medication. (OM and FBT *p* = 0.01). No association was found between adverse reactions and dosage of opioids used for BTP (*p* = 0.78).

## 5. Factors Influencing BTP Clinical Presentation

### 5.1. Number of BTP Episodes/Day

Males, old patients, and patients with higher Karnofsky levels, had a higher number of episodes/day (*p* = 0.00, *p* = 0.04, and *p* = 0.00, respectively). Distribution of the number of episodes of BTP/day among the different setting was irregular: patients admitted in hospice or home care had more episodes in comparison with inpatients or outpatients (*p* = 0.00), Patients with nociceptive pain and predictable BTP had a higher number of BTP episodes/day (*p* = 0.00). Patients with colon-rectal cancer and esophageal cancer, had a lower number of BTP episodes (*p* = 0.01 and *p* = 0.00 respectively), while patients with head and neck cancer and pancreatic cancer had a higher number of BTP episodes/day (*p* = 0.00 and *p* = 0.01, respectively). Patients with fast onset BTP had a higher number of BTP episodes/day (*p* = 0.00). There was a weak correlation with background pain intensity (Pearson correlation coefficient: 0.3, *p* = 0.00).

Data of multivariate analysis is presented in Table 2. Age, head and neck cancer, higher Karnofsky levels, background pain intensity, predictable BTP, and fast onset were independently associated with a higher number of BTP episodes.

### 5.2. Intensity of BTP

At the univariate analysis, variables associated with a higher BTP intensity were younger age (*p* = 0.00), in-hospital place of visit, outpatient, and day-hospital visit (*p* = 0.00), neuropathic and mixed pain (*p* = 0.00), lung cancer and urological cancer (*p* = 0.00), grade of oral mucositis (*p* = 0.02), and background pain intensity (*p* = 0.01), BTP intensity was lower in colon-rectal cancer (*p* = 0.05), liver (*p* = 0.04, breast cancer (*p* = 0.02) and fast onset BTP (*p* = 0.00). Table 3 shows the results of multivariate analysis for BTP intensity.

In the multivariate analysis, BTP pain intensity was independently associated with higher background pain intensity, fast onset BTP, and lower level of Karnofsky.

### 5.3. Predictability of BTP

At the univariate analysis, variables associated with predictable BTP were older age (*p* = 0.00), outpatient and in-hospital places of visit (both *p* = 0.00), pain mechanism (*p* = 0.00, mixed and nociceptive) head and neck cancer (*p* = 0.00), breast cancer (*p* = 0.05), grade or oral mucositis (*p* = 0.00), low intensity of BTP and fast onset of BTP (*p* = 0.00 and 0.05, respectively), and loco-regional disease (*p* = 0.03). Pancreatic cancer and gastric cancer were associated with unpredictable BTP (*p* = 0.02 and 0.03, respectively). Table 4 shows the results of multivariate analysis for BTP predictability. Pain mechanism (neuropathic pain) was independently associated with unpredictable BTP (*p* = 0.00).

### 5.4. Time to Maximum BTP Intensity (BTP Onset)

At the univariate analysis, variables associated with fast-onset BTP were day-hospital and in-hospital places of visit (*p* = 0.00), pain mechanism (mixed and nociceptive) (*p* = 0.00), predictable BTP (*p* = 0.05), colon-rectal cancer (*p* = 0.05), disease-oriented anticancer treatment (*p* = 0.00), radiotherapy, targeted therapy and chemotherapy, targeted therapy (*p* = 0.00), grade of mucositis (G2–G4) (*p* = 0.00). The slow onset was associated with gynecological cancer and breast cancer (*p* = 0.02 and *p* = 0.01, respectively). Variables independently associated with slow onset were lower Karnofsky Performance Scale (KPS), gynecological and breast cancer, no anticancer treatment, nociceptive background pain, and unpredictable BTP (Table 5).

### 5.5. Duration of Untreated BTP Episodes

A long duration of BTP was associated with old age (*p* = 0.00), hospice or home setting (*p* = 0.00), nociceptive background pain (*p* = 0.00), predictable BTP (*p* = 0.00), pancreatic cancer (*p* = 0.00), prostate cancer (*p* = 0.00), and disease-oriented anticancer treatment (*p* = 0.00). BTP duration was inversely related to the background pain intensity, the higher background pain intensity, the shorter BTP duration (*p* = 0.00). Head and neck and gastric cancer were associated with a short duration of BTP (*p* = 0.003 and 0.003, respectively). Table 6 shows the results of multivariate analysis for BTP duration. Variables independently associated with a longer duration of BTP were: age, place of visit (home > hospice > day-hospital > outpatients, inpatients) (*p* = 0.00), and some type of cancers, disease-oriented therapy, background pain intensity and mechanism, and unpredictable BTP.

### 5.6. Interference of BTP with Daily Activities

Variables associated with interference of BTP with daily activities were: younger age (less interference, *p* = 0.00), Karnofsky (less interference, *p* = 0.00), gender (male, more interference, *p* = 0.04), places of visit (day-hospital and in-hospital had higher level of interference in comparison with hospice, (*p* = 0.00), pain mechanism (mixed and nociceptive, higher level of interference, *p* = 0.00), unpredictable BTP (higher level of interference, *p* = 0.00). Brain, breast cancer, and sarcoma were associated with little or no interference (*p* = 0.02, *p* = 0.00 and *p* = 0.02, respectively), Lung cancer was associated with higher level of interference (*p* = 0.01). Much or very much interference with life activities were associated with fast onset BTP (*p* = 0.00), metastatic disease (*p* = 0.04), disease-oriented anticancer treatment (*p* = 0.01), and oral mucositis (*p* = 0.00).

The level of interference was directly proportional to background pain intensity (*p* = 0.00), and duration of BTP (*p* = 0.00). Table 7 shows the results of multivariate analysis of factors interfering with daily activities. Age, Karnofsky, background pain intensity, fast onset and long duration of BTP were independently associated with interference with daily activity.

## 6. Discussion

To our knowledge, this is the largest survey currently available of BTP in cancer patients. The only existing study of BTP in such a large sample was performed in a US population of commercially insured community-dwelling patients, predominantly non-cancer, with opioid-treated chronic pain [18]. Information gathered from this study provided a new step in the knowledge of the phenomenon of BTP in cancer patients, underlining the complex interactions among a series of factors influencing the clinical presentation.

This study indicates that the BTP may have different characteristics and that treatment should be set on the individual patient’s condition. A diagnostic algorithm [10,11] based on a clear distinction between background pain and BTP, pointed out that the difference between background pain intensity and BTP intensity was more than 3 points on a 0 to 10 numerical scale, thus ensuring a clear distinction between background pain and BTP for most patients. This information also helps preventing possible misinterpretations of data, as occurred in previous studies where background pain intensity and BTP intensity were not clearly distinguished [5]. Of interest, adverse reactions attributed to BTP medications were reported in a small number of patients and were mostly mild in intensity, and not related to BTP medication doses. 

### 6.1. Factors Influencing BTP Presentation

#### 6.1.1. Number of BTP Episodes/Day

A mean number of four BTP episodes/day is commonly considered acceptable [3]. In this study, only a minority of patients presented ≥5 episodes per day. Some independent variables were identified to be associated with more episodes/day of BTP. Patients with higher levels of activity are more likely to develop episodes of BTP, particularly with an incident component (predictable pain) with a rapid onset. Interestingly, patients who had ≥5 episodes of BTP/day were more likely to have predictable and fast-onset BTP, possibly induced by movement. These relationships are commonly observed in a clinical setting. On the other hand, higher background pain intensity, despite being within the range of so-called controlled pain (mild pain, 0–4 on a numerical scale) may favor the development of BTP episodes, confirming previous observations. It has been reported that severe background pain intensity was shown to be a powerful predictor of BTP scores [19,20]. In many epidemiological studies of BTP, background analgesia was not optimal and was based on non-opioid or weak opioids medications [20,21,22,23,24]. This finding confirms that a better background analgesia may decrease the number of BTP episodes [9], and allow a better patient mobilization [5]. This suggests the need to optimize background analgesia to limit the number of BTP episodes [25], particularly in patients with bone metastases, and their intensity (see paragraph below).

#### 6.1.2. Intensity of BTP

Younger patients with higher background pain, a fast onset and predictable BTP, and a lower Karnofsky level develop higher BTP intensities. A rapid development of BTP is likely to produce a high intensity of pain. Pain expression is more important in younger patients, possibly because of their psychological distress [26], as well in patients with head and neck cancer. Similarly, patients with worse clinical conditions may have a greater disease burden.

In this study, BTP intensity was well distinguished from background pain intensity (about three points difference on average), suggesting that patients with BTP were well selected, according to a predefined algorithm. In previous European studies, the percentages of patients with severe BTP were 57–61% only, and often a distinction between BTP and background pain intensities was unclear [10,16,27].

#### 6.1.3. Predictable BTP

Predictable BTP has obvious implications in terms of prevention and therapeutic interventions. Physical activity and swallowing were the most frequent factors triggering BTP episodes. The percentage of patients with predictable BTP was consistent with previous epidemiological studies [11,16]. In a previous analysis, with a limited number of patients, predictable BTP was more likely observed in certain disease-related conditions (primary tumor, setting, anticancer treatments) and in patients with lower background pain and BTP intensities [15]. Patients with bone metastases, who are more often observed in radiotherapy setting, may have lower pain levels at rest. The lower BTP intensity in patients with predictable BTP might be explained, for example, by stopping a movement that triggers BTP in case of bone metastases [5]. In fact, patients with predictable BTP were more likely to report a non-pharmacological relieving factor [10,16]. The BTP predictability in the presence of oral damage could be easier to explain, as in the presence of mucositis, swallowing typically evokes BTP. However, in this study, the only relevant finding was that patients with a neuropathic pain component were more likely to have unpredictable BTP. Consequently, patients with a prevalent nociceptive component, for example bone metastases, tend to develop predictable episodes. 

#### 6.1.4. Time to Maximum BTP Intensity (Onset)

Time to maximum BTP intensity has obvious clinical implications for a timely therapeutic intervention and possible psychological input in asking for a medication [27]. A dichotomous measure was chosen for distinguishing fast-onset from slow-onset BTP (≤10 or >10 min, respectively). In multicenter European surveys [10,11,16], time to maximum BTP intensity onset was 10–15 min, but no further analyses were conducted to identify risk factors. In a preliminary analysis of a limited number of patients, a fast-onset BTP was associated with serious mucositis or radiotherapy setting [15]. This can be explained by the immediate pain input by a damaged mucosa, for example on swallowing. Moreover, a fast-onset BTP is typical in patients with pain on movement due to bone metastases [10], who are often referred to radiotherapy. Indeed, BTP developed more slowly when BTP intensity was lower or in patients with a lower Karnofsky status. Thus, the risk of fast or slow-onset BTP could be characterized according to these conditions. In this study, representing the pattern of a large number of patients, a lower Karnofsky level, gynecological and breast cancer, no anticancer treatment, nociceptive background pain, and unpredictable BTP were independently associated with a slow onset of BTP. One could argue that more advanced patients, no longer receiving anticancer treatment, develop BTP slowly and in an unpredictable way, possibly because their limited activity, that is they are more likely to have an idiopathic BTP, not triggered by known causes. Other features regarding certain types of cancer deserve further analysis and should be better explored.

#### 6.1.5. Duration of Untreated BTP Episodes

The mean duration of untreated BTP episodes was similar to that described in previous surveys, reporting a variable duration of 30–60 min [10,11,15,16,19,20,21,22]. Indeed, duration of untreated episodes is based on patients’ recalling and difficult to be properly assessed by patients. Thus, interpretation of this data should be cautious.

Some factors were independently associated with the duration of BTP. A longer duration of BTP episodes was found in hospice and home care patients, as well as in older patients, in previous studies, patients who had a low background pain intensity, or who were admitted to hospice or were seen at home, had a longer BTP duration [11,15]. Of interest, predictable BTP was less likely to be associated with a longer BTP duration. As reported before, one could argue that patients with predictable pain, due to an incident component for example, could stop their precipitating activity and pain can spontaneously disappear [5], differently from patients with idiopathic BTP, not triggered by any known precipitating factor [16]. The finding that nociceptive was independently associated with a longer BTP duration should deserve further investigation, as in this category somatic and visceral pain could be differently represented. Finally, some cancers and disease-oriented treatments were more likely to be associated with a different BTP duration. These aspects deserve further analyses. 

#### 6.1.6. Interference of BTP with Daily Activities

The presence of BTP limited patients’ daily life, particularly in younger patients and in patients with a lower Karnofsky status and higher background pain intensity. These findings are clinically understandable, although never reported in literature. Younger patients may have more psychological distress [26], and patients with worse clinical conditions may have a greater disease burden. Of course, higher background pain, even within the level of so-called controlled pain (≤4/10) is commonly associated with larger interference with daily life activities. More importantly, a fast-onset BTP and a longer duration were also associated with notable interference with daily activities. This means that the faster and the longer BTP event, the worse interference exists.

## 7. Conclusions

The data gathered from the large sample of patients included in this study revealed that BTP is a multifaceted phenomenon. This study suggests that physicians should investigate the presence of BTP in every patient with cancer pain. In addition, BTP therapy should be calibrated on the clinical condition and characteristics of BTP. The identification of risk factors for the development of specific subtypes of BTP may allow better personalizing treatments according to individual clinical features. Further studies have been planned to assess some variables associated with certain care settings, disease types, and anticancer therapies.

## Figures and Tables

**Table 1:** Characteristics of patients.

**Age (Years) Mean (SD)**		64.6 (12.24), Range 18–97 Years
**Gender (M/F)**		2202 (54.8%)/1814 (45.2%)
**Karnofsky mean (SD)**		61.8 (18.73), range 10–100
**Primary tumor**	Lung	1089 (27.1%)
	Gastrointestinal	647 (16.1%)
	Breast	480 (11.9%)
	Pancreas	349 (8.7%)
	Urological	242 (6.0%)
	Prostate	224 (5.6%)
	Head-neck	205 (5.1%)
	Gynecologic	185 (4.6%)
	Liver	135 (3.4%)
	Haematological	98 (2.4%)
	Others	511 (12.7%)
**Disease**	Loco-regional	642 (16.0%)
	Metastatic	3374 (84.0%)
**Anticancer treatment**	Disease-oriented	3030 (78.0%)
	Palliative Care	854 (22.0%)
**Place of Visit **	Outpatients	1378 (34.3%)
	Day hospital	462 (11.5%)
	Home care	577 (14.4%)
	Hospice	101 (2.5%)
	Hospital inpatient	1498 (37.3%)
**Setting**	Palliative care	720 (17.9%)
	Oncology	2087 (52.0%)
	Pain therapy	1184 (29.5%)
	Radiotherapy	25 (0.6%)
**Mean background pain intensity at T0**		3.0 (SD 1.83)
**Mean opioid doses (expressed as oral morphine equivalents)**		69.4 mg/day (SD 88.7) mg/day

**Table 2:** Multivariate analysis for the number of BTP episodes/day.

Factors	β	*p*	(95% CI)	
Age	0.05	0.03	0.00	0.87
Gender	−0.00	0.90	−0.01	0.00
Karnofsky	0.01	0.05	−0.01	0.00
Setting	−0.00	1.00	−0.02	0.02
Colon-rectal cancer	−0.03	0.38	−0.09	0.04
Head and neck	0.13	0.00	0.05	0.23
Pancreatic cancer	0.02	0.54	−0.05	0.09
Esophageal cancer	0.17	0.07	−0.01	0.34
Background pain intensity	0.15	0.00	0.12	0.16
Type of BTP (predictable)	−0.21	0.00	−0.24	−0.16
Onset	−0.16	0.00	−0.20	−0.11

**Table 3:** Multivariate analysis for BTP intensity.

Factors	β	*p*	(95% CI)	
Age	−0.01	0.09	−0.02	0.00
Place of visit	0.00	0.84	−0.01	0.01
Karnofsky	−0.01	0.00	−0.02	0.00
Grade of mucositis	0.02	0.30	−0.01	0.03
Colon-rectal cancer	−0.01	0.70	−0.04	0.03
Liver	−0.03	0.36	−0.10	0.04
Breast	−0.01	0.50	−0.05	0.03
Lung	0.00	0.51	−0.02	0.04
Urological	0.03	0.36	−0.03	0.07
Disease-oriented anticancer treatment	−0.01	0.57	−0.04	0.02
Background pain intensity	0.04	0.00	0.03	0.51
Type of BTP (predictable)	0.01	0.10	−0.01	0.03
Time to maximum BTP intensity	−0.04	0.00	−0.06	−0.01

**Table 4:** Multivariate analysis for predictable BTP.

Factors	β	*p*	(95% CI)	
Age	−0.01	0.37	−0.02	0.00
Place of visit	0.00	0.63	−0.03	0.02
Karnofsky	0.00	0.82	−0.01	0.00
Head and neck cancer	−0.05	0.43	−0.17	0.07
Breast	−0.04	0.39	−0.11	0.04
Pancreas	0.04	0.38	−0.05	0.12
Gastric	0.06	0.39	−0.07	0.18
Metastatic disease	0.03	0.49	−0.04	0.09
Grade of mucositis	−0.02	0.25	−0.07	0.02
Background pain intensity	0.01	0.34	−0.01	0.03
Mechanism of Background pain	0.05	0.00	0.01	0.07
Time to maximum BTP intensity	0.02	0.36	−0.03	0.07

**Table 5:** Multivariate analysis for BTP slow onset.

Factors	OR	*p*	(95% CI)	
Place of visit	0.02	0.47	−0.03	0.07
Karnofsky	−0.01	0.00	−0.01	0.00
Colon-rectal cancer	−0.16	0.15	−0.38	0.06
Gynecological	0.38	0.01	0.07	0.69
Breast	0.32	0.00	0.12	0.53
Anticancer treatment	−0.31	0.00	−0.47	−0.14
Grade of mucositis	0.08	0.18	−0.04	0.20
Mechanism of Background pain	−0.11	0.00	−0.19	−0.03
Type of BTP	0.23	0.00	0.08	0.39

**Table 6:** Multivariate analysis for duration of untreated BTP episodes.

Factors	β	*p*	(95% CI)	
Age	0.01	0.00	0.00	0.02
Place of visit	0.09	0.00	0.08	0.10
Karnofsky	0.00	0.51	−0.01	0.00
Head & neck cancer	−0.41	0.00	−0.46	−0.36
Pancreas	0.38	0.00	0.36	0.41
Gastric	−0.39	0.00	−0.45	−0.33
Prostate	0.25	0.00	0.22	0.29
Disease-oriented anticancer treatment	0.38	0.00	0.35	0.40
Background pain intensity	−0.11	0.00	−0.12	−0.10
Background pain mechanism (nociceptive)	−0.08	0.00	−0.09	−0.07
Unpredictable BTP	0.55	0.00	0.53	0.56

**Table 7:** Multivariate analysis of factors interfering with daily activities.

Factors	β	*p*	(95% CI)	
Age	−0.01	0.03	−0.02	0.00
Karnofsky	−0.01	0.00	−0.02	−0.01
Gender	0.03	0.20	−0.02	0.08
Place of visits	−0.02	0.21	−0.03	0.01
Brain	−0.33	0.17	−0.79	0.14
Breast	−0.04	0.37	−0.11	0.04
Lung	0.01	0.96	−0.05	0.05
Sarcoma	−0.01	0.92	−0.18	0.16
Disease-oriented anticancer treatment	0.02	0.48	−0.03	0.04
Disease	0.01	0.81	−0.05	0.06
Grade of mucositis	0.02	0.54	−0.03	0.05
Background pain intensity	0.05	0.00	0.02	0.07
Mechanism of Background pain	0.01	0.28	−0.01	0.04
Type of BTP	0.01	0.89	−0.04	0.05
Time to maximum BTP intensity	−0.05	0.03	−0.10	0.00
Duration of untreated BTP	0.01	0.00	0.00	0.02

## Data Availability

The datasets generated during/and or analyzed during the current study are available from the corresponding author on request.
